# Surface reactivity of amphibole asbestos: a comparison between crocidolite and tremolite

**DOI:** 10.1038/s41598-017-14480-z

**Published:** 2017-10-31

**Authors:** Giovanni B. Andreozzi, Alessandro Pacella, Ingrid Corazzari, Maura Tomatis, Francesco Turci

**Affiliations:** 1 Dipartimento di Scienze della Terra, Sapienza Università di Roma, Piazzale Aldo Moro 5, I-00185 Roma, Italy; 2 CNR-IGG, U.O. Roma, c/o Dipartimento di Scienze della Terra, Sapienza Università di Roma, Piazzale Aldo Moro 5, I-00185 Roma, Italy; 3 Dipartimento di Chimica, Università di Torino, via Pietro Giuria 7, I-10125 Torino, Italy; 4 “G. Scansetti” Interdepartmental Centre for Studies on Asbestos and Other Toxic Particulates, Università di Torino, via Pietro Giuria 9, I-10125 Torino, Italy

## Abstract

Among asbestos minerals, fibrous riebeckite (crocidolite) and tremolite share the amphibole structure but largely differ in terms of their iron content and oxidation state. In asbestos toxicology, iron-generated free radicals are largely held as one of the causes of asbestos malignant effect. With the aim of clarifying i) the relationship between Fe occurrence and asbestos surface reactivity, and ii) how free-radical generation is modulated by surface modifications of the minerals, UICC crocidolite and fibrous tremolite from Maryland were leached from 1 day to 1 month in an oxidative medium buffered at pH 7.4 to induce redox alterations and surface rearrangements that may occur in body fluids. Structural and chemical modifications and free radical generation were monitored by HR-TEM/EDS and spin trapping/EPR spectroscopy, respectively. Free radical yield resulted to be dependent on few specific Fe^2+^ and Fe^3+^ surface sites rather than total Fe content. The evolution of reactivity with time highlighted that low-coordinated Fe ions primarily contribute to the overall reactivity of the fibre. Current findings contribute to explain the causes of the severe asbestos-induced oxidative stress at molecular level also for iron-poor amphiboles, and demonstrate that asbestos have a sustained surface radical activity even when highly altered by oxidative leaching.

## Introduction

Asbestos is a group of six naturally-occurring silicate encompassing one fibrous serpentine (chrysotile) and five fibrous amphiboles (anthophyllite, tremolite, actinolite, riebeckite, and grunerite, with the last two commercially known as crocidolite and amosite, respectively). Asbestos have been widely used in the past in a number of industrial settings and are now banned in several countries, due to health risks posed by their inhalation^1^. Despite an extraordinary research effort, the mechanism on the basis of their toxicity is still partially unclear. Indeed, asbestos toxicity resides on a complex combination of physico-chemical properties and, among these, surface reactivity related to the presence and bioavailability of Fe has received considerable attention by the scientific community^2–4^. Iron ions were considered to play a key role in asbestos toxicity because of their ability to catalyse generation of reactive oxygen species (ROS)^5–7^. Also, the formation of ferruginous bodies, mainly made of iron, are still investigated to disentangle the complex interactions between asbestos and human lung, in the context of asbestos toxicology^8–12^. However, the specific structural, coordinative, and oxidative states that make surface iron a reactive surface site involved in free radical generation are not yet fully elucidated. This is largely due to the intrinsic variability of fiber surface that may undergo chemical modifications when in contact with biological fluids, with consequent modification of the iron topochemistry that may result in the modulation of the fibre reactivity. Mineral surface is indeed a dynamic entity that dialogues with biological media, cells and tissues^13^, and may be altered by the surrounding media in a very complex way. Exposure of chrysotile to acidic bio-weathering, for example, promotes the incongruent leaching of brucitic layer, in which iron substitutes for magnesium, and reduces the surface reactivity^14^. Amphiboles are more stable than serpentine asbestos in acidic solutions^15^ and are less susceptible to chemical modifications following interaction with body fluids^16^. Nevertheless, previous researches showed that a few hour leaching of crocidolite and tremolite with an oxidative buffered medium caused a significant modification of the Fe^2+^/Fe^3+^ ratio in the surface layers and the occurrence of newly-formed iron-rich phases^17,18^. These studies highlighted a different behaviour of crocidolite with respect to tremolite in both dissolution and iron speciation kinetics. Iron-rich crocidolite exhibited the fastest dissolution of the external layers with the strong promotion of bulk iron to the surface and consequent precipitation of Fe-rich nanoparticles. Iron-poor tremolite showed a sluggish dissolution process with partial amorphisation of external layers and absence of armouring. To understand how these complex surface dynamics modulate the overall surface reactivity of asbestos and determine the nature of the molecular interaction between asbestos fibres and biological molecules, this work investigates i) the surface reactivity of crocidolite and tremolite asbestos, measured as the ability to catalyse free radical release, and ii) how this parameter is affected by the surface alterations induced by an oxidative leaching. Crocidolite and tremolite fibres were incubated in a H_2_O_2_ solution buffered at pH 7.4 for different time points, from 1 day to 1 month, under the same conditions used in the previous studies^17,18^. Far from mimicking the cellular environment, such conditions have been chosen to promote the dissolution dynamics of asbestos in a reasonable experimental time. Structural and chemical evolution of leached fibres were followed by high resolution transmission electron microscopy (HR-TEM) and energy dispersive spectroscopy (EDS). Free radical release was investigated by spin trapping technique coupled with electron paramagnetic resonance (EPR) spectroscopy considering two biologically relevant reactions known to likely occur *in vivo*: the Fenton reaction of H_2_O_2_ with surface iron producing hydroxyl radical (HO^•^) and the surface-driven homolytic cleavage of the C-H bond in formate anion yielding a carbon-centred radical (COO^−•^)^19^.

## Results

### HR-TEM analysis of leached amphibole asbestos

**Figure 1:**
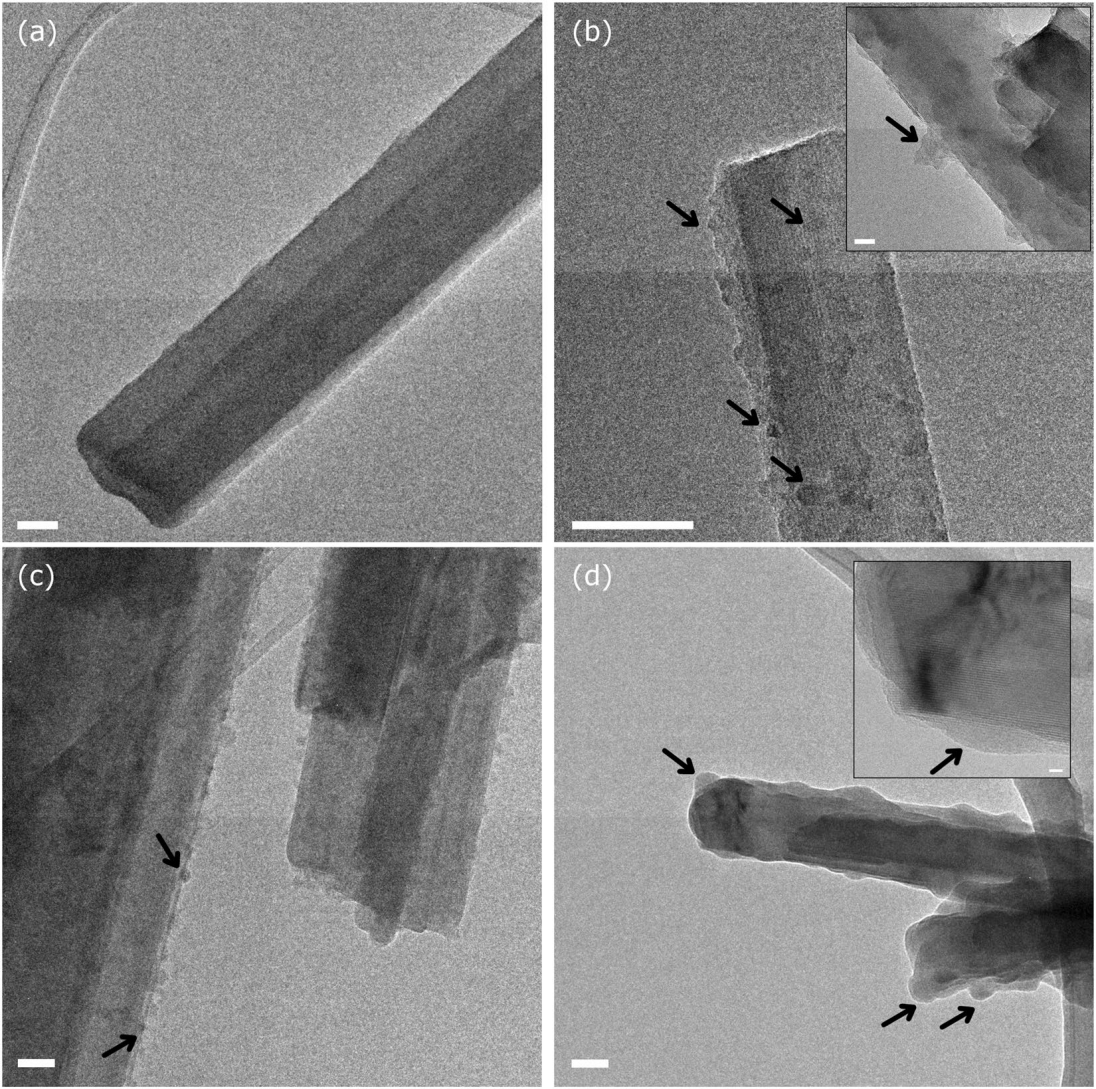
Representative HR-TEM images of pristine and 24, 48 and 720 hour leached UICC crocidolite standard (**a**,**b**,**c**, and **d**, respectively). Arrows in (**b**) and (**c**) highlight neo-formed iron-rich nanoparticles, in (**d**) point to large patches of amorphous material. Relative scale bars: 50 nm, main pictures; 5 nm, high-resolution insets.

**Figure 2:**
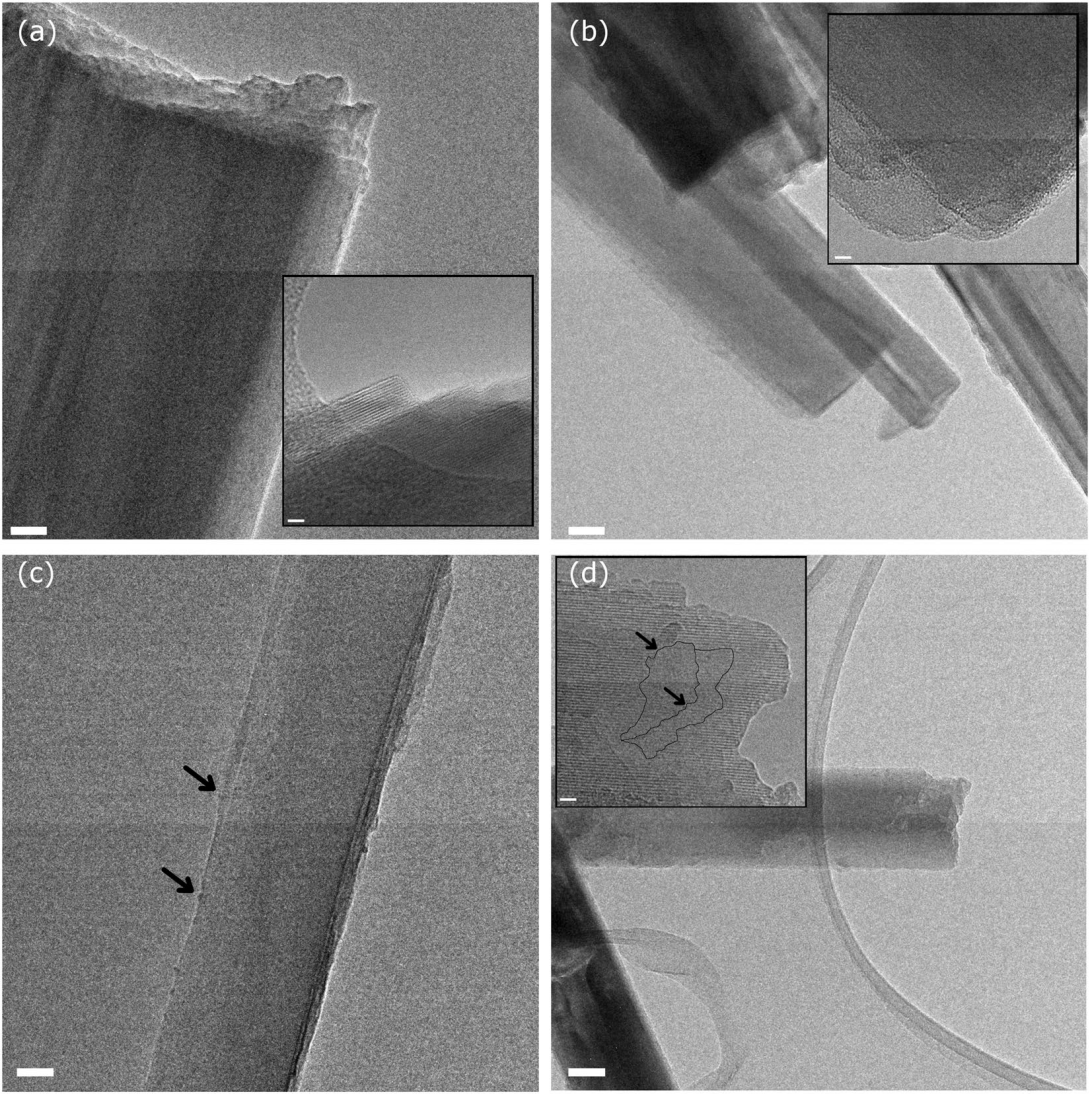
Representative HR-TEM images of pristine and 24, 48 and 720 hour leached tremolite from Maryland (**a**,**b**,**c**, and **d**, respectively). Insets in (**a**) and (**b**) evidence higly-ordered crystalline lattice. Arrows in (**c**) indicate sporadic, incipient formation of altered nanometric clusters; in (**d**) point to large patches of amorphous layers. Relative scale bars: 50 nm, main pictures; 5 nm, high-resolution insets.

Results obtained from HR-TEM investigation on pristine (untreated) crocidolite and tremolite fibres (Figs 1 and 2, panels *a*) are presented in comparison with those obtained on the same fibres incubated in H_2_O_2_/KPB at pH 7.4 for 24 h, 48 h, and 1 month (Figs 1 and 2, panels *b*, *c*, and *d*, respectively). HR-TEM analysis of the same fibres incubated for one week in identical conditions are reported in previous works^17,18^ and are here comparatively discussed. Both pristine amphiboles showed long and thin fibres with micrometric to nanometric width (≥100 nm) consisting of associated single-crystal nanometric fibrils, which revealed the perfect amphibole growth along *c* axis (Figs 1 and 2, panels *a*).

TEM analysis of the leached crocidolite revealed an early alteration of the outermost layers of the fibres already after 24 hours of leaching. Neo-formed nanoparticles were also observed (Fig. 1b, arrows). EDS analysis highlighted a slight increase of Fe/Si ratio when X ray spectrum is collected on the nanoparticles (Supplementary Information, Fig. S1). After 48h-leaching, crocidolite was further degraded and several nanoparticles were present on the fibre surface (Fig. 1c). EDS analysis suggested that Fe, Ca, and K phosphates might have concurred in forming such particles (Supplementary Information, Fig. S2). No evidence of the presence of iron-rich accessory phases was found. This is consistent with previous results^17^ that showed the precipitation of Fe– and Ca–phosphates onto fibres after 1-week leaching experiment. Large patches of amorphous material could be detected after 1 month of leaching (Fig. 1d, arrows). Lattice defects and/or stalking faults may be observed as dark zones at higher magnification (Fig. 1d, inset) Even if stacking faults and defects can be common in amphiboles, the frequency of defects on leached fibres appeared to be higher than pristine sample. Also, discontinuity in the diffraction fringes of mineral crystal planes highlighted the discontinuity between crystal lattice of crocidolite and the neo-formed amorphous precipitate (Fig. 1d, inset, arrow).

TEM images of pristine tremolite evidenced a highly-ordered crystalline lattice (Fig. 2a), and diffraction fringes from defect-free crystallographic planes could be observed at high-resolution (Fig. 2a, inset). Following exposure to the oxidative medium, Maryland tremolite showed an alteration kinetics much slower than crocidolite. The tremolite structure resulted virtually unaltered after 24 and 48 hours of leaching (Fig. 2b and c). The incipient formation of few altered nanometric clusters was occasionally observed on the 48 h-leached sample, while discontinuity in diffraction fringes, signalling large patches of surface-grown amorphous areas, were found after 1 week (data in previous work^18^) and after 1 month leaching time (Fig. 2d, inset).

### Free radical generation activity of the pristine fibres

To assess the surface reactivity of the investigated amphiboles, two well-known tests were carried out, namely the production of hydroxyl radicals (HO^•^) via Fenton reaction (Equation 1), and the production of carboxyl radicals (COO^−•^) via the homolytic cleavage of the C-H bond in formate anion H-COO^−^ (Equation 2):

1 $${{{\rm{Fe}}}^{2+}}_{{\rm{surf}}}+{{\rm{H}}}_{2}{{\rm{O}}}_{2}\to {{{\rm{Fe}}}^{3+}}_{{\rm{surf}}}+{{\rm{OH}}}^{-}+{{\rm{HO}}}^{\bullet }$$

2 $${{{\rm{Fe}}}^{2+}}_{{\rm{surf}}}+{{\rm{H}} \mbox{-} \mathrm{COO}}^{-}\to {{{\rm{Fe}}}^{3+}}_{{\rm{surf}}}+{{\rm{COO}}}^{-\bullet }+{{\rm{H}}}^{+}$$

The release of the hydroxyl radical in the presence of hydrogen peroxide is held to occur *in vivo* when asbestos fibres are exposed to lysosomal fluids during alveolar macrophage phagocytosis, promoting a direct oxidative stress. The reaction may take place also in the presence of ferric iron (Fenton-like reaction, Equations 3a and 3b), that can be reduced to ferrous iron in a non-acidic medium^20,21^:

3a $${{\rm{Fe}}}^{3+}+{{\rm{H}}}_{2}{{\rm{O}}}_{2}\leftrightarrow {\rm{Fe}}\cdots {{\rm{OOH}}}^{2+}+{{\rm{H}}}^{+}$$

3b $${\mathrm{Fe} \mbox{-} \mathrm{OOH}}^{2+}\to {{\rm{Fe}}}^{2+}+{{\rm{HO}}}_{2}^{\bullet }$$

The homolytic cleavage of the C-H bond in formate anion was employed as a model reaction that may occur to several biomolecules including peptides, proteins and lipids. Such a reaction yields the formation of a carbon-centred radical COO^−•^ (Equation 2) and, for chrysotile asbestos, was reported to be strongly dependent on the presence of ferrous iron at the fibre surface^22^. Since natural samples are exposed to oxidative chemical weathering, iron at the fibre surface likely occurs as ferric ion^5^. In presence of a reducing agent (Equation 4), such as ascorbic acid, the radical production is usually triggered by promoting surface iron reduction:

4 $${{{\rm{Fe}}}^{3+}}_{{\rm{surf}}}+{{\rm{Asc}}}^{-}\to {{{\rm{Fe}}}^{2+}}_{{\rm{surf}}}+{{\rm{Asc}}}^{\bullet }$$

**Figure 3:**
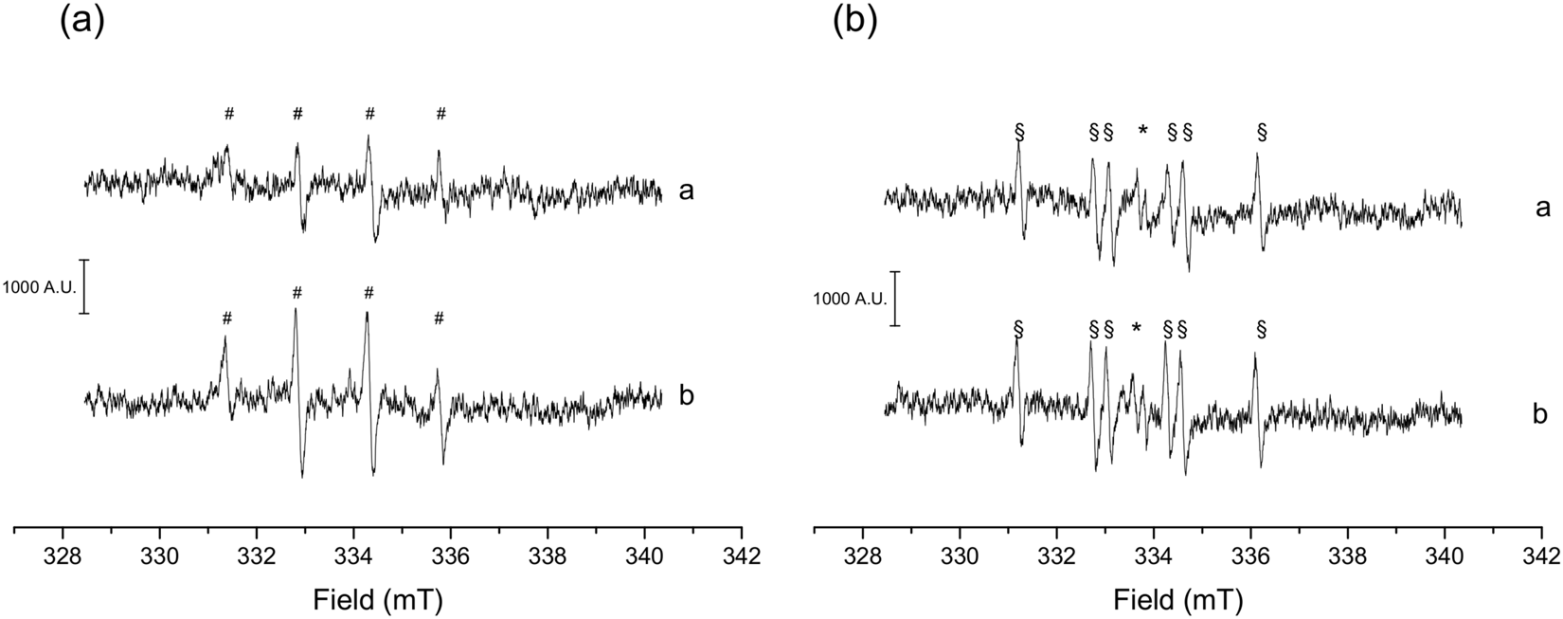
Representative EPR spectra of (**a**) [DMPO-OH]^•^ and (**b**) [DMPO-COO^−^]^•^ adduct (# and §, respectively) obtained from (a) pristine crocidolite and (b) pristine tremolite fibres recorded after 60 min of incubation with H_2_O_2_ and HCOO^−^ as target molecules, respectively. Ascorbyl radical (*) was observed in b as the reaction mixture was added with ascorbic acid as reducing agent.

The surface-generated radicals were stabilized with the spin-trapping (DMPO), and EPR spectra of [DMPO-HO]^•^ (Fig. 3a) and [DMPO-COO^−^]^•^ (Fig. 3b) adducts were recorded. To quantify the amount of radical generated, each EPR spectrum was double-integrated and the total amount of radicals released during 1 h of incubation of the asbestos fibres with the target molecule was considered (Supplementary Information, Tables S1 and S2). Both pristine amphiboles are active in generating HO^•^ and COO^−•^. However, crocidolite proved to be slightly less reactive than tremolite in HO^•^ release (Fig. 3a, spectra *a* and *b*, respectively). Interestingly, COO^−•^ generation was observed only in presence of ascorbic acid (Fig. 3b). Due to the presence of ascorbic acid, the EPR signal of the ascorbyl radical, as intermediate specie in the oxidation of ascorbic acid to dehydroascorbic acid, was detected (Fig. 3b, * mark).

### Alteration of the free radical generation activity upon leaching

**Figure 4:**
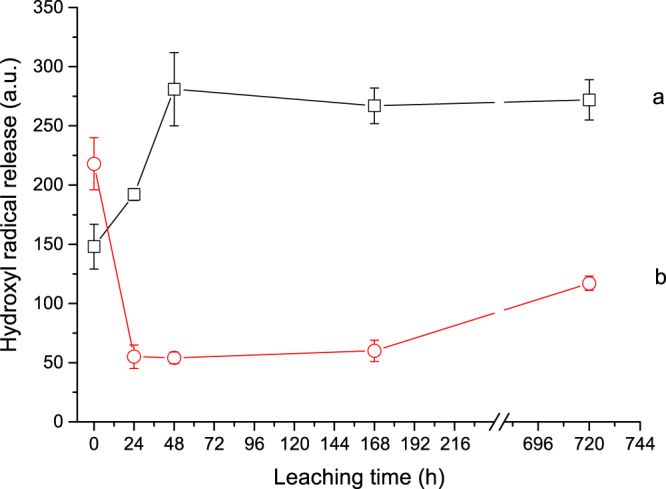
Total [DMPO-OH]^•^ production of the investigated asbestos samples as a function of the leaching time (0, 24, 48, 168, 720 h): (a) UICC crocidolite standard, (b) Maryland tremolite.

The amount of HO^•^ radicals released by the samples leached in the oxidative medium for 24, 48, 168 and 720 hours is reported in Supplementary Information (Tables S1 and S2) and displayed in Fig. 4. For leached crocidolite, the reactivity was significantly higher than that of the pristine fibres: in more detail, the radical production suddenly increased with leaching time in the range 0–48 h, and no further increase was observed when additional leaching was implemented up to 1 month (Fig. 4a). Conversely, the surface reactivity of the long-leached tremolite samples was lower than that of the pristine one: in fact, a significant and abrupt decrease of the hydroxyl radical production was observed in the first 24 hours followed by a slow recovery up to 1 month (Fig. 4b).

**Figure 5:**
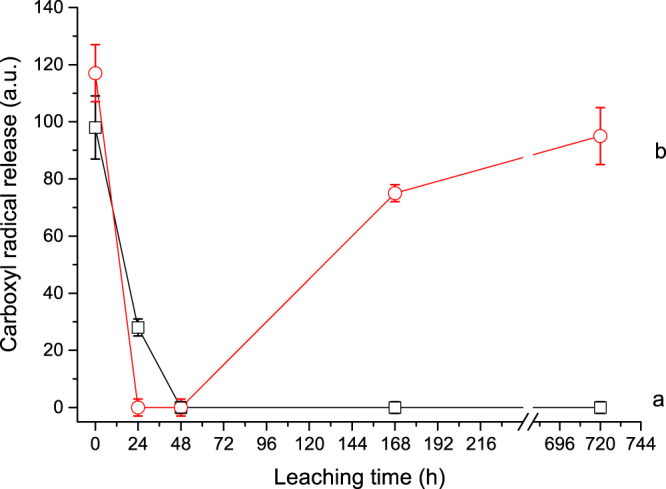
Total [DMPO-COO^−^]^•^ production of the investigated asbestos samples in presence of ascorbic acid as a function of the incubation time (0, 24, 48, 168, 720 h): (a) UICC crocidolite standard, (b) Maryland tremolite.

The amount of COO^−•^ radicals produced upon leaching is reported in Fig. 5. Data refer to reactivity in the presence of ascorbic acid because, as already observed for pristine fibres, COO^−•^ radicals were not released when tests were performed in a non-reducing medium. This behaviour was previously observed for other asbestos fibres^23^ and is probably due to the oxidation state of surface iron by chemical weathering. Even in presence of ascorbic acid, for crocidolite the COO^−•^ radical release was rapidly suppressed upon leaching (Fig. 5a). The radical yield was measurable only for the pristine and 24 h-leached fibres and was negligible from 48h-leaching time onwards. Similarly, tremolite exhibited the maximum COO^−•^ radical yield in its pristine, unaltered surface state, followed by a sudden decrease to zero after 24 h. However, for this sample a consistent increase of the radical yield to higher values occurred after 48 h, which was gently sustained up to the longest leaching time (Fig. 5b).

## Discussion

Among amphibole asbestos, crocidolite is one of the richest in iron. The UICC sample contains almost ten times Fe ions per formula unit with respect to Maryland tremolite. A large difference in iron content (up to four times, measured by XPS) was also evidenced for Fe ions occurrence at surface^17,18^. The two samples also strongly differ in the Fe oxidation state, with about 50% of total Fe in the ferric oxidation state for the crocidolite against less than 20% for the tremolite. In spite of that, surfaces of both of them are prevalently oxidized, with more than 70% of the surface Fe in the ferric oxidation state, according to XPS results^24^.

The EPR/spin-trapping analysis of the iron-related radical reactivity of the two fibres shows that tremolite is slightly more reactive than crocidolite in both HO^•^ and COO^−•^ production (the latter measured in presence of ascorbic acid). This supports the hypothesis that the free-radical surface reactivity in asbestos is neither related to the total iron content, nor to the amount of iron on the surface, but it is likely due to specific iron sites in a well-defined coordination and oxidative state^19,22,25^.

**Figure 6:**
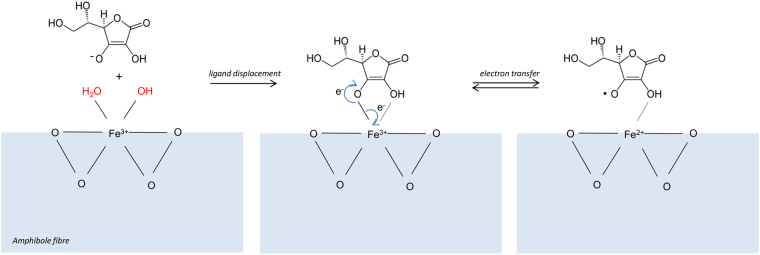
Reductive electron transfer between ascorbate and octahedral surface Fe^3+^. The adventitious coordinative positions of surface iron (in red) are removed via a ligand displacement mechanism. Only iron centres with at least two free coordinative positions may react with ascorbic acid. An electron is transferred from ascorbate to ferric iron, that is reduced to ferrous iron. This latter may consequently be involved in the homolytic cleavage of C-H bond in formate anion, and the paramagnetic ascorbyl radical is evidenced by EPR spectroscopy.

The observed behaviour of both fibres, which were not able to induce cleavage of the C-H bond in H-COO^−^ in absence of ascorbic acid, is a first confirmation of the above mentioned hypothesis. This behaviour indicates that the vast majority of iron ions exposed at the very first surface atomic layer – which is much thinner than the “surface” probed by spectroscopic surface techniques such as XPS – is likely oxidized (or in any case unable to participate to a redox cycle). The ferric form is indeed thermodynamically favoured by the oxidative action of chemical weathering, and is definitely compatible with previous findings^24^. The presence of ascorbic acid as reducing agent in the H-COO^−^ reaction mixture promptly reactivated the Fe^3+^ ions through a well-known electron transfer mechanism (Equation 4 and Fig. 6). In both crocidolite and tremolite structure, iron has an octahedral coordination with six ligands represented by either six oxygen atoms (of the double chains of silica tetrahedra that sandwich the layers containing the cations) or four oxygen atoms and two hydroxyl anions^26^. These six structural ligands are partially lost when surface discontinuity interrupts the bulk structure^13^. The unoccupied coordinative valences of iron are, in air or aqueous media, usually occupied by molecular water or hydroxyl groups that can be replaced by stronger ligand, such as iron chelators including ascorbic acid^25^. Due to steric and conformational reasons, only a surface iron ion with at least two unoccupied coordinative valences may effectively interact with ascorbic acid, via its hydroxyl groups in α- and β- position on the lactone ring^27^. When ligand substitution occurs, electron transfer may take place and surface low-coordinated ferric ions may be reduced to ferrous ions and become reactive in the homolytic cleavage of the C-H bond in formate anion (Fig. 6).

Upon leaching, the iron-rich surface of UICC crocidolite underwent quick alteration, as indicated by TEM analysis and fully discussed in a previous study on dissolution kinetics^17^. Reactivity data are interpreted supposing that, after 24/48 h of leaching, the first atomic layer of crocidolite surface is rapidly deprived of low-coordinated iron ions^25^ –which are the less thermodynamically stable– and the oxidative leaching medium rapidly promotes the oxidation of Fe^2+^ to Fe^3+^. With respect to pristine crocidolite, after 24/48 h of leaching the fibre surface is indeed enriched with high-coordinated Fe^3+^ (i.e., ferric ions bearing only one coordinative position not occupied by structural ligands). The generation of COO^−•^ radical indeed requires either Fe^2+^ or low-coordinated Fe^3+^ for ascorbic acid reduction to take place, while hydrogen peroxide used in the HO^•^ test may reduce high-coordinated Fe^3+^ since just one free coordinative position is sufficient for the reductive electron transfer to take place^28^. The reduced ferrous iron in turn promotes the degradation of another hydrogen peroxide molecule to hydroxyl radical, a Fenton-like multi-step reaction^20^, which likely accounts for the observed increase in HO^•^ yield. This oxidative and coordinative evolution of iron explains the dramatic changes observed for the crocidolite reactivity over time, with the HO^•^ radical generation increasing to almost double and the COO^−•^ dropping to zero after 24/48 h of leaching (Figs 4a and 5a). For longer leaching time points, TEM analysis evidenced the presence of Fe-oxide and phosphate nanoparticles precipitated on the altered surface (Fig. 1). This armouring virtually neither masked the active sites nor contributed to reactivity, since we observed that both HO^•^ and COO^−•^ radical production are not affected. This is consistent with the amphibole alteration model recently defined by some of us^18^, that proposes the establishing of a steady state between the fast dissolution kinetics of external layer and surface iron speciation. The neo-formed iron phosphate and oxy-hydroxide nanoparticles are poorly reactive in redox cycles and do not modulate the overall crocidolite radical reactivity due to the high nuclearity of the Fe centres and the stability of the structural coordinative ligands^29–31^.

Tremolite exhibited a remarkably different behaviour upon leaching with respect to crocidolite mostly due to their different crystal chemistry, that is the presence of Ca and Mg (instead of Na and Fe^2+^) in the B and C amphibole crystallographic sites that provide a more stable structure^17,18^. This may explain why tremolite surface underwent minor chemical and structural modification with respect to crocidolite, as evidenced by TEM images (Fig. 2 vs. Fig. 1). In spite of that, at the earliest stage of the oxidative leaching also for tremolite a complete suppression of the COO^−•^ radical production was observed (Fig. 5b). This is likely caused by prompt substitution of high-coordinated ions for the low-coordinated redox reactive iron ions within the first 24 hours. At the same leaching step, a parallel decrease of the HO^•^ radical production (though never zero) was recorded for tremolite (Fig. 4b). This behaviour is opposite to that displayed by crocidolite and is likely due to the low number of iron sites at the tremolite surface^18^. Nevertheless, the residual reactivity observed for HO^•^ confirms that high-coordinated iron, which is less prone to be removed from the surface, is co-responsible for radical reactivity towards hydrogen peroxide, but is inactive towards formate. Longer leaching time (>48 h) enhanced the tremolite radical reactivity towards hydrogen peroxide and restored the reactivity towards formate anion. This behaviour is consistent with tremolite sluggish dissolution kinetics, that is in turn responsible for the partial surface amorphisation observed by TEM (Fig. 2, see also previous work^18^ for further details). In fact, dissolution of the bulk promotes the relatively few Fe^2+^ ions to surface, producing low-coordinated Fe^3+^ (reducible by ascorbic acid and active in the test towards formate). Moreover, the neo-formed amorphous layer likely stabilises low-coordinated iron ions and prevent their aggregation, the latter feature being essential for sustaining the radical reactivity (especially the COO^−•^ production) for longer time.

Our results on amphibole asbestos are in agreement with previous results on iron-doped synthetic chrysotile^22,32^ and evidence that the highest reactivity, and possibly toxicity, in asbestos is associated with the occurrence of low-coordinated, low-nuclearity iron sites.

## Conclusions

Pathogenic-related surface reactivity of fibrous amphiboles, crocidolite and tremolite, has been demonstrated to be dependent on specific surface Fe sites rather than the total Fe content of the minerals. Both ferrous iron and ferric iron with one or more unsaturated coordinative valences are involved in the Fenton and Fenton-like reactions with hydrogen peroxide, which yield HO^•^ radical. Both ferrous iron and ferric iron with two or more unsaturated coordinative bonds (this latter only after reaction with ascorbic acid) are responsible for the production of COO^−•^ radical from formate anion.

Results obtained studying the evolution of reactivity during leaching up to one month (in oxidative medium buffered at pH 7.4) demonstrate that amphiboles have a sustained surface radical activity even when highly altered by oxidative leaching. Tremolite, in particular, exhibits a significant radical production from both pristine and altered fibres.

The experimental approach here adopted and results obtained can be helpful in shedding light on the biologically-relevant reactivity of asbestos ferruginous bodies that resides in human lung for decades^8–12^.

## Methods

### Chemical composition of the studied fibrous samples

The two fibrous samples investigated in this work were: crocidolite (that is the finely fibrous variety of amphibole riebeckite) standard by the Union Internationale Contre le Cancer (UICC); a tremolite specimen from Montgomery County, Maryland (USA). The tremolite was collected in a serpentinite from an ophiolite sequence belonging to the Piney Branch Complex, southern part of the Appalachian Ophiolitic Complex (extending from Virginia to Pennsylvania), which consists of highly altered peridotite, pyroxenite, and gabbro^33^.

Empirical structural formula of the UICC crocidolite standard was obtained by coupling ICP-OES and Mössbauer spectroscopy data^24^:

^B^(K_0.01_Na_1.64_Ca_0.14_Mg_0.16_)_Σ=1.95_^C^(Fe^2+^_2.13_Fe^3+^_2.30_Mg_0.55_Mn_0.01_Ti_0.01_)_Σ=5.00_^T^(Si_7.82_Al_0.02_)_Σ=7.84_ O_22_^O3^(OH)_2.1_, fairly close to that of the endmember riebeckite ^B^Na_2_^C^(Fe^2+^_3_Fe^3+^_2_)_Σ=5.00_^T^Si_8_O_22_^O3^(OH)_2_.

The full chemical, structural and spectroscopic characterization of the fibrous tremolite from Maryland was carried out by optimizing EMP, XRPD and Mössbauer spectroscopy data^33^. Empirical structural formula is:

^B^(Ca_1.99_Na_0.01_Mn_0.02_)_Σ2.02_^C^(Mg_4.48_Fe^2+^_0.44_Mn_0.02_Fe^3+^_0.08_^VI^Al_0.01_)_Σ5.03_^T^(Si_7.95_Al_0.01_)_Σ7.96_

O_22_^O(3)^(OH_1.97_F_0.01_)_Σ1.98_, fairly close to that of the endmember tremolite ^B^Ca_2_^C^Mg_5_^T^Si_8_O_22_^O3^(OH)_2_.

The error on both the EMP and ICP analysis is ±1 wt% relative on the major elements (wt% >1), corresponding up to ±0.05 atoms per formula unit, and up to ±10 wt% relative on the minor elements (0.02 < wt% < 0.1), corresponding up to ± 0.005 atoms per formula unit.

### Fibre incubation in oxidative buffered solution at pH 7.4

Asbestos fibres were incubated for 24 h, 48 h, 168 h (1 week) and 720 h (1 month). Experimental conditions of fibre leaching were those reported in previous works^17,18^. Specifically, 25 mg of fibres was suspended in 2 ml of a hydrogen peroxide (0.1 M) solution buffered with potassium phosphate (KPB, 0.5 M, pH 7.4). The suspensions were continuously shaken in a thermostatic oscillating bath at 37 °C. To recover the fibres from the tubes, the samples were centrifuged at 10000 × g for 10 min and then rinsed with 2 ml of Milli-Q ultrapure deionised water for 3 times prior to reactivity tests. If not stated differently, all reagents were from Sigma-Aldrich (Milan, Italy). Ultrapure Milli-Q water (Merck-Millipore, Vimondrone, Italy) was used throughout.

### HR-TEM investigation

The structural and morphological alteration induced on the two fibrous amphiboles by leaching in the hydrogen peroxide buffered solution were monitored after 24 h, 48 h and 1 month. Characterisation of pristine and incubated samples after one week are reported in previous works^17,18^. Samples were investigated by JEOL 3010-UHR High-Resolution Transmission Electron Microscopy (HR-TEM) with a LaB_6_ filament operated at 300 KeV, beam current 114 µA and equipped with a 2 K × 2 K pixels Gatan US1000 CCD camera. Elemental analysis was performed by Oxford INCA X-ray energy dispersive spectrometer (X-EDS) with a Pentafet Si(Li) detector. The fibres were dispersed in ultrapure MilliQ water, briefly sonicated to improve particle dispersion and deposited on Lacey Carbon Cu grids.

### EPR measurement of free radical production

The potency of both pristine and incubated samples to generate free radicals was evaluated using Electron Paramagnetic Resonance (EPR) spectroscopy following a well-established procedure^19^. All tests were performed at 37 °C in absence of light. The spectra were recorded on a Miniscope MS 100 (Magnettech, Berlin, Germany) CW-EPR spectrometer. Instrument settings were as follows: microwave power, 10 mW; modulation, 1 G; scan range, 120 G; centre of field, approximately 3345 G. Experiments were performed at least in duplicate and a blank procedure was always carried out. To quantify the amount of radical generated, each EPR spectrum was double-integrated as described in previous works^19,22^. The area of the energy adsorption peak was adopted as arbitrary measure for the total amount of radicals released during 1 h of incubation of the asbestos fibres with the target molecule (H_2_O_2_ or formate anion).

#### HO^•^ radical generation

The reaction tube contained 25 mg of sample, 500 µl of 0.5 M potassium phosphate buffer (pH 7.4) and 250 µl of DMPO (5,5-dimethyl-1-pyrroline-N-oxide, Enzo Life Sciences, Inc., NY, USA) as spin trapping agent (0.18 M). The reaction was triggered adding 250 µl of H_2_O_2_ (0.2 M). The tube was placed on a magnetic stirrer to ensure the homogeneity of the suspension. A fraction of the suspension was drawn after 10, 30, and 60 minutes, filtered through cellulose acetate (0.25 µm porosity) membranes and then transferred into a 50 μl-capillary tube for EPR measurements.

#### COO^−•^ radical generation

The reaction mixture contained 25 mg of sample, 250 µl of DMPO (0.18 M), 500 µl of sodium formate (1 M) in 0.5 M phosphate buffer (pH 7.4) and 250 µl ultrapure MilliQ water or ascorbic acid (3 mM) as reductive agent. Experiments were performed as specified above.

## Electronic supplementary material

Supplementary Info

## References

[1] International Agency for Research on Cancer (IARC). In *Arsenic*, *metals*, *fibres*, *and dusts* Vol. 100C *Monographs on the evaluation of carcinogenic risks to humans* 219–309 (World Health Organization (WHO), 2012). PMC4781271 23189751

[2] Shukla A (2003). Multiple roles of oxidants in the pathogenesis of asbestos-induced diseases. Free Radical Biol. Med..

[3] Manning CB, Vallyathan V, Mossman BT (2002). Diseases caused by asbestos: mechanisms of injury and disease development. Int. Immunopharmacol..

[4] Fubini B, Arean CO (1999). Chemical aspects of the toxicity of inhaled mineral dusts. Chem. Soc. Rev..

[5] Hardy JA, Aust AE (1995). Iron in asbestos chemistry and carcinogenicity. Chem. Rev..

[6] Kamp DW (2009). Asbestos-induced lung diseases: an update. Transl.Res..

[7] Liu, G., Cheresh, P. & Kamp, D. W. Molecular Basis of Asbestos-Induced Lung Disease. *Annual Review of Pathology: Mechanisms of Disease*, **Vol 8 8**, 161–187, 10.1146/annurev-pathol-020712-163942 (2013). 10.1146/annurev-pathol-020712-163942 PMC3900296 23347351

[8] Ghio AJ, Churg A, Roggli VL (2004). Ferruginous bodies: implications in the mechanism of fiber and particle toxicity. Toxicol. Pathol..

[9] Bardelli F (2017). New insights on the biomineralisation process developing in human lungs around inhaled asbestos fibres. Sci Rep.

[10] Pascolo, L. *et al*. Synchrotron soft X-ray imaging and fluorescence microscopy reveal novel features of asbestos body morphology and composition in human lung tissues. *Particle and Fibre Toxicology* **8**, 10.1186/1743-8977-8-7 (2011). 10.1186/1743-8977-8-7 PMC3041679 21299853

[11] Pascolo L (2013). The interaction of asbestos and iron in lung tissue revealed by synchrotron-based scanning X-ray microscopy. Sci Rep.

[12] Bursi Gandolfi, N., Gualtieri, A. F., Pollastri, S., Tibaldi, E. & Belpoggi, F. Assessment of asbestos body formation by high resolution FEG-SEM after exposure of Sprague-Dawley rats to chrysotile, crocidolite, or erionite. *J. Hazard. Mater.* **306**, 95–104 (2016). 10.1016/j.jhazmat.2015.11.050 26705886

[13] Hochella MF (1993). Surface chemistry, structure, and reactivity of hazardous mineral dust. Reviews in Mineralogy and Geochemistry.

[14] Turci F (2007). A biomimetic approach to the chemical inactivation of chrysotile fibres by lichen metabolites. Chemistry-A European Journal.

[15] Virta, R. L. Asbestos: geology, mineralogy, mining, and uses. Report No. 2002-149, (US Geological Survey., 2002).

[16] Committee on asbestos: selected health effects - Institute of Medicine (US). *Asbestos**:**Selected Cancers*. (National Academies Press 2006). 20669440

[17] Pacella A (2014). Dissolution reaction and surface iron speciation of UICC crocidolite in buffered solution at pH 7.4: A combined ICP-OES, XPS and TEM investigation. Geochim. Cosmochim. Acta.

[18] Pacella A (2015). Surface alteration mechanism and topochemistry of iron in tremolite asbestos: A step toward understanding the potential hazard of amphibole asbestos. Chem. Geol..

[19] Fubini B, Mollo L, Giamello E (1995). Free radical generation at the solid/liquid interface in iron containing minerals. Free Radical Res..

[20] Ensing B, Buda F, Baerends EJ (2003). Fenton-like chemistry in water: Oxidation catalysis by Fe(III) and H2O2. J. Phys. Chem. A.

[21] Walling C, Goosen A (1973). Mechanism of the ferric ion catalyzed decomposition of hydrogen peroxide. Effect of organic substrates. J. Am. Chem. Soc..

[22] Turci F, Tomatis M, Lesci IG, Roveri N, Fubini B (2011). The iron-related molecular toxicity mechanism of synthetic asbestos nanofibres: A model study for high-aspect-ratio nanoparticles. Chemistry - A European Journal.

[23] Tomatis M, Prandi L, Bodoardo S, Fubini B (2002). Loss of surface reactivity upon heating amphibole asbestos. Langmuir.

[24] Fantauzzi M (2010). Combined use of X-ray photoelectron and Mossbauer spectroscopic techniques in the analytical characterization of iron oxidation state in amphibole asbestos. Anal. Bioanal. Chem..

[25] Martra G, Tomatis M, Fenoglio I, Coluccia S, Fubini B (2003). Ascorbic acid modifies the surface of asbestos: possible implications in the molecular mechanisms of toxicity. Chem. Res. Toxicol..

[26] Hawthorne FC, Oberti R (2007). Amphiboles: Crystal chemistry. Amphiboles: Crystal Chemistry, Occurrence, and Health Issues.

[27] Suter D, Banwart S, Stumm W (1991). Dissolution of Hydrous Iron(Iii) Oxides by Reductive Mechanisms. Langmuir.

[28] Graf E, Mahoney JR, Bryant RG, Eaton JW (1984). Iron-catalyzed hydroxyl radical formation. Stringent requirement for free iron coordination site. J. Biol. Chem..

[29] Fierro G, Moretti G, Ferraris G, Andreozzi GB (2011). A Mössbauer and structural investigation of Fe-ZSM-5catalysts: Influence of Fe oxide nanoparticles size on the catalytic behaviour for the NO-SCR by C3H8. Appl. Catal., B.

[30] Moretti G, Fierro G, Ferraris G, Andreozzi GB, Naticchioni V (2014). N2O decomposition over [Fe]-MFI catalysts: Influence of the FexOy nuclearity and the presence of framework aluminum on the catalytic activity. J. Catal..

[31] Freyria FS (2012). Hematite nanoparticles larger than 90 nm show no sign of toxicity in terms of lactate dehydrogenase release, nitric oxide generation, apoptosis, and comet assay in murine alveolar macrophages and human lung epithelial cells. Chem. Res. Toxicol..

[32] Gazzano E (2007). Iron-loaded synthetic chrysotile: A new model solid for studying the role of iron in asbestos toxicity. Chem. Res. Toxicol..

[33] Ross, M. & Nolan, R. P. In *Ophiolite concept and the evolution of geological thought* (eds Yildirim Dilek & Sally Newcomb) (Geological Society of America, 2003).

