# Supplementary material for: Surface reactivity of amphibole asbestos: a comparison between crocidolite and tremolite
Source: Sci Rep. 2017 Oct 31;7:14696. doi: 10.1038/s41598-017-14480-z (PMC5665974; doi:10.1038/s41598-017-14480-z)
Supplement: Supplementary file 1 — Supplementary Info [file 41598_2017_14480_MOESM1_ESM.pdf]

Surface reactivity of amphibole asbestos: a comparison between crocidolite and tremolite

Giovanni B. Andreozzi[1,2], Alessandro Pacella[1], Ingrid Corazzari[3,4], Maura Tomatis[3,4], and Francesco Turci[3,4,*]

[1] Dipartimento di Scienze della Terra, Sapienza Università di Roma, Piazzale Aldo Moro 5, I-00185 Roma, Italy

[2] CNR-IGG, U.O. Roma, c/o Dipartimento di Scienze della Terra, Sapienza Università di Roma, Piazzale Aldo Moro 5, I-00185 Roma, Italy

[3] Dipartimento di Chimica, Università di Torino, via Pietro Giuria 7, I-10125 Torino, Italy

[4] "G. Scansetti" Interdepartmental Centre for Studies on Asbestos and Other Toxic Particulates, Università di Torino, via Pietro Giuria 9, I-10125 Torino, Italy

Supporting information

Table S1. Intensity of the [DMPO-HO]$^{\bullet}$ obtained for the investigated samples. Means and standard deviations (in brackets) were calculated over two measurements

	UICC crocidolite				Maryland tremolite			
	10 min	30 min	60 min	**total**	10 min	30 min	60 min	**total**
**pristine**	61 (7)	51 (3)	36 (8)	**148 (19)**	67 (9)	81 (4)	70 (9)	**218 (22)**
**24h**	60 (1)	60 (1)	73 (3)	**192 (5)**	-	20 (3)	35 (7)	**55 (10)**
**48h**	82 (11)	87 (1)	111 (20)	**281 (32)**	-	21 (2)	34 (2)	**55 (4)**
**168h**	109 (2)	81 (10)	77 (4)	**267 (15)**	-	22 (7)	38 (2)	**60 (9)**
**720h**	72 (2)	85 (0)	114 (15)	**272 (17)**	24 (3)	42 (2)	51 (1)	**117 (6)**

Table S2. Intensity of the [DMPO-COO^{-}]$^{\bullet}$ adduct obtained for the investigated samples. Means and standard deviations (in brackets) were calculated over two measurements

	UICC crocidolite				Maryland tremolite			
	10 min	30 min	60 min	**total**	10 min	30 min	60 min	**total**
**pristine**	-	32 (2)	66 (9)	**98 (11)**	22 (3)	29 (3)	66 (4)	**117 (10)**
**24h**	-	-	28 (3)	**28 (3)**	-	-	-	**-**
**48h**	-	-	-	**-**	-	-	-	**-**
**168h**	-	-	-	**-**	-	34 (1)	41 (2)	**75 (3)**
**720h**	-	-	-	**-**	20 (3)	31 (7)	44 (1)	**95 (11)**

Figure S1 – EDS analysis of pristine and 24h leached UICC crocidolite

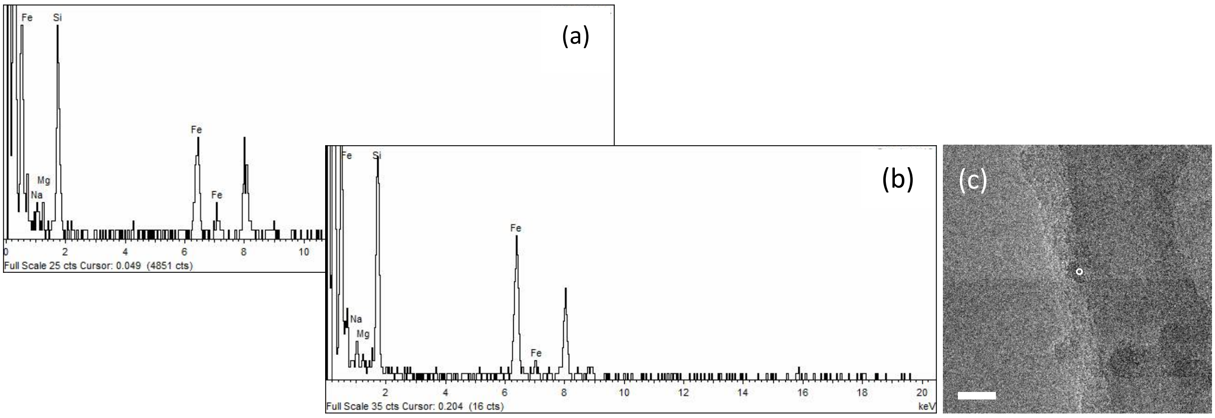

The EDS spectra of UICC crocidolite, pristine (a) and leached for 24h (b). Some darker areas, indicating the occurrence of elements with higher electronic density, were observed (c, relative scale bar 5 nm) and associated to the presence of neo-formed phases of iron-rich precipitates. EDS analyses were performed on these darker spots (e.g., white circle) with a spot size resolution of about 25 nm. Quantitative results highlighted an increased Fe/Si ratio of about 5 at.% with respect to the average pristine crocidolite composition (spectrum *a*) when X-ray spectrum is collected on these iron-rich nanoparticles. Unlabeled peaks at ca. 8 keV are the Kα1 and Kα2 X-ray lines of Cu from TEM sample holder.

Figure S2 – EDS analysis of neoformed particles on 48h-leached UICC crocidolite surface

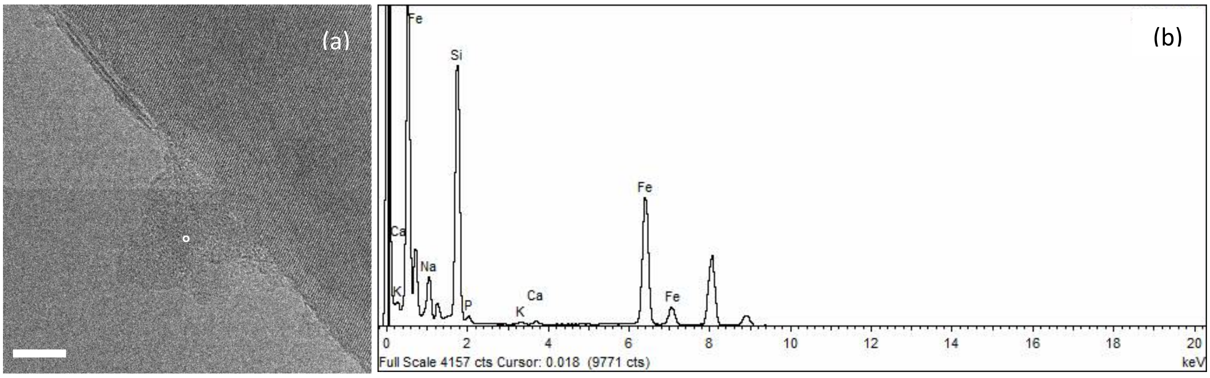

HR-TEM imaging and EDS analysis of the 48h-incubated UICC crocidolite. Several iron-rich nanoparticles were observed on the fibre surface (a, relative scale bar 5 nm). EDS analysis (b) evidenced the occurrence of P, Ca, and K, suggesting that iron, calcium and potassium phosphates might have concurred in forming such particles. The discontinuity between crocidolite fibres and the neoformed particles as well as the amorphous nature of such particles are evidenced by the absence of the fringes due to crocidolite lattice diffraction. Unlabeled peaks at ca. 8 keV are the Kα1 and Kα2 X-ray lines of Cu from TEM sample holder.
